# Involvement of the kynurenine pathway in breast cancer: updates on clinical research and trials

**DOI:** 10.1038/s41416-023-02245-7

**Published:** 2023-04-11

**Authors:** Hemaasri-Neya Girithar, Ananda Staats Pires, Seong Beom Ahn, Gilles J. Guillemin, Laurence Gluch, Benjamin Heng

**Affiliations:** 1 Faculty of Medicine, Health and Human Sciences, Macquarie University, Sydney, NSW Australia; 2 The Strathfield Breast Centre, Strathfield, NSW Australia

**Keywords:** Metabolomics, Breast cancer

## Abstract

Breast cancer (BrCa) is the leading cause of cancer incidence and mortality in women worldwide. While BrCa treatment has been shown to be highly successful if detected at an early stage, there are few effective strategies to treat metastatic tumours. Hence, metastasis remains the main cause in most of BrCa deaths, highlighting the need for new approaches in this group of patients. Immunotherapy has been gaining attention as a new treatment for BrCa metastasis and the kynurenine pathway (KP) has been suggested as one of the potential targets. The KP is the major biochemical pathway in tryptophan (TRP) metabolism, catabolising TRP to nicotinamide adenine dinucleotide (NAD^+^). The KP has been reported to be elevated under inflammatory conditions such as cancers and that its activity suppresses immune surveillance. Dysregulation of the KP has previously been reported implicated in BrCa. This review aims to discuss and provide an update on the current mechanisms involved in KP-mediated immune suppression and cancer growth. Furthermore, we also provide a summary on 58 studies about the involvement of the KP and BrCa and five clinical trials targeting KP enzymes and their outcome.

## Breast cancer

**Fig. 1:**
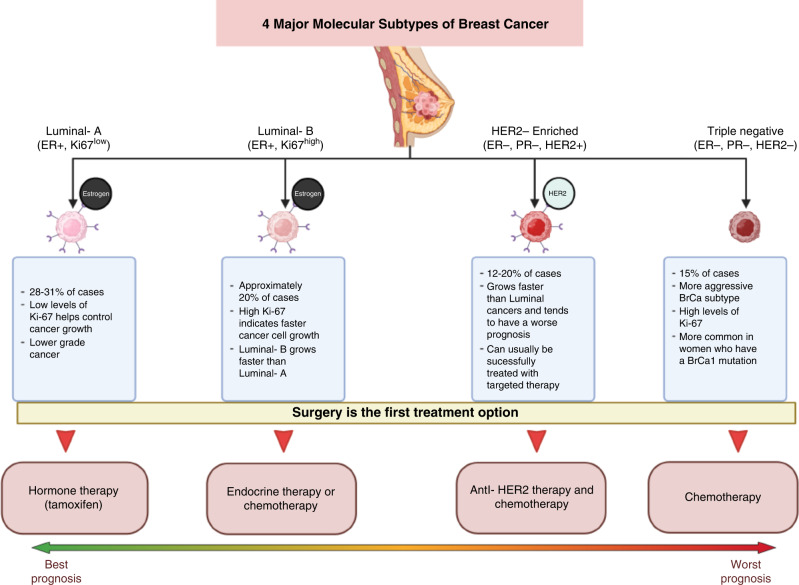
Schematic diagram of major BrCa subtypes and its clinical profiles. BrCa can be categorised into four major subtypes based on its molecular characteristics: Luminal-A, luminal-B, HER2-enriched and TNBC. Up to 30% and 20% of total BrCa cases are luminal-A and luminal-B subtype, respectively. Both subtypes have the highest survival rate of up to 85% due to the availability of targeted treatments. HER2-enriched subtype accounts for 20% of total cases and has a lower 5-year survival rate of 75%. Finally, it is the TNBC that makes up to 15% of total cancer cases and has the poorest 5-year survival rate of 62%. This is due to the absence of targeted treatments that the other subtypes have.

Breast cancer (BrCa) is the leading cause of cancer incidence worldwide with 2.26 million cases in 2020 alone or 11.7% of total cancer cases [1]. It is also the fourth leading cause of cancer-related deaths with 685,000 deaths in 2020 or 6.9% of total cancer-related death [1]. Although BrCa leads cancer incidence worldwide, it is generally curable in a majority of diagnosed patients [1, 2]. This is partly due to a combination of early detection and advances in treatment [1, 3]. Treatment of BrCa is usually guided by molecular characteristics of the tumour [4]. Four of the most common subtypes [5] include: luminal-A [6], luminal-B [7, 8], human epidermal growth factor receptor 2 (HER2)-enriched [9] and triple-negative (TNBC) [10] as summarised in Fig. 1. Luminal-A and B subtypes [11] have the highest 5-year cancer survival rates followed by HER2-enriched [9, 12], and TNBC [13].

Despite the combination of advancements in early screening and detection and targeted therapy as highlighted above, metastasis remains a cause of more than 90% of BrCa deaths [14]. Immunotherapy has emerged as one of the cancer treatment options to contain or slow down the spread of cancer [15]. One treatment approach involves the boosting of the host’s suppressive ability against cancer cells as demonstrated by immune checkpoint inhibitors including anti-cytotoxic T-lymphocyte associated antigen-4 (CTLA-4) and programmed death -1 (PD-1; pembrolizumab) and PD ligand-1 (PD-L1; atezolizumab) [16]. While these treatments have improved overall survival in patients with metastatic melanoma [17] and non-small cell lung cancer[18], the overall response rates to immunotherapy with pembrolizumab or atezolizumab in metastatic TNBC BrCa are still modest (between 5% and 25% [19–21]. Combinatory regimens of these immune checkpoint inhibitors and chemotherapy are currently under investigation as detailed in the review by Thomas et al. [22]. The modest response rates in TNBC BrCa to immune checkpoint inhibitors implies the involvement of different immune evasion mechanisms. The KP has been suggested as the alternate mechanism that tumour cells can use to suppress local immune surveillance to facilitate growth and spread [23].

## The KP

TRP is one of the essential amino acids that is not endogenously synthesised and needs to be obtained from the diet for protein synthesis [24]. Once introduced with food, TRP is absorbed from the gut and released into the bloodstream, where it is transported to various tissues and cells [25]. The diet-derived TRP has distinct metabolic fates - while up to 30% of TRP and a smaller portion of TRP is used as a precursor for protein synthesis [26] and the production of neurotransmitter serotonin and neuromodulator/neurohormone melatonin respectively, most TRP is metabolised through the KP [27] (Fig. 1). One of the main physiological roles of the KP is to generate NAD + through the KP. Three different enzymes can catabolise TRP into the KP first metabolic step: tryptophan 2,3 dioxygenase (TDO) [28]; indoleamine 2,3 dioxygenase 1 (IDO1) [29] and indoleamine 2,3 dioxygenase 2 (IDO2) [30–32]. These enzymes catalyse the same enzymatic step, although each of them has distinct inducers and patterns of tissue expression.

TDO is expressed mainly in the liver [33] and other selected organs such as brain [34], embryonic tissues [35] and placenta [36]. TDO is activated by its substrate TRP or by glucocorticoids [37]. TDO can be inhibited by oestrogens and progesterone [38], as well as by NAD/NADH by a negative feedback loop [39]. IDO1 is expressed virtually in all major organs [40] and monocyte lineage immune cells [41–43]. The transcription and expression of IDO1 is rapidly upregulated by pro-inflammatory cytokines such as interferon-γ (IFN-γ) tumour necrotic factor-α (TNF-α) [44] and other pro-inflammatory mediators, such as lipopolysaccharide, amyloid peptides, and viral proteins among others [45]. Conversely, IDO1 is inhibited by its substrate TRP [46], by anti-inflammatory cytokines such as interleukin (IL)-10 and IL-4, as well as by the antioxidant enzyme superoxide dismutase [47]. IDO2 expression is detected in neuronal cells of the cerebral cortex, hepatocytes, the bile duct and dendritic cells [48, 49]. IDO2 is a less well-studied enzyme, and its inducer and biological roles remain unclear [30–32].

**Fig. 2:**
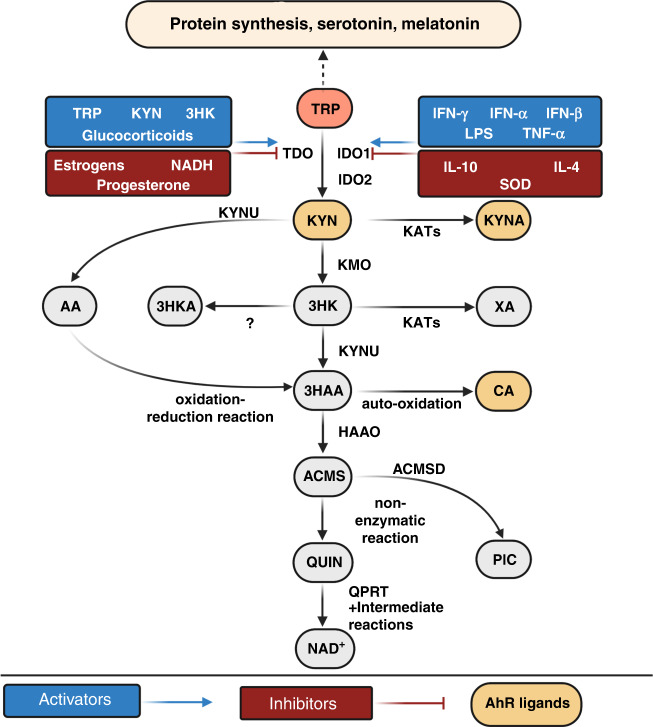
Schematic representation of kynurenine pathway metabolites production. TRP is a precursor for protein synthesis and neurotransmitter serotonin and neuromodulator/neurotransmitter melatonin. The majority of the dietary TRP is catabolised through the KP. TDO, one of the rate-limiting enzymes of the pathway, is activated by elevated glucocorticoids, KYN, 3HK and TRP and inhibited by NADH. IDO is another rate-limiting enzyme of the KP, and it is activated in the presence of pro-inflammatory mediators such as TNF-α, IFN-γ, IFN-α, IFN-β and LPS. On the other hand, IDO is inhibited by SOD and anti-inflammatory mediators such as IL-4 and IL-10. Following the KP activation, TRP is metabolised into metabolites through specific enzymes catalysing each reaction in various cells/tissues. KYN, a central metabolite of the KP and can be catabolized into three different intermediates, KYNA, AA and 3HK by KATS, KYNU and KMO respectively. 3HK can further be converted to 3HAA by KYNU and then to 2-amino-3-carboxymuconate semialdehyde ACMS by HAAO. Alternatively, 3HK can also be metabolised by KATs into XA or into 3HKA. Additionally, the oxidation-reduction reaction can convert AA to 3HAA, while 3HAA by auto-oxidation can produce CA. The metabolite ACMS is positioned at a key junction of the pathway to either non-enzymatically form the metabolite QUIN, a precursor for *de novo* synthesis of NAD^+^ or converted to PIC by ACMSD.

As mentioned above, TRP can be metabolised into the KP producing NAD^+^ product, or into other KP intermediates in a multi-branched arrangement, mediated by enzymes and their inducers acting at the different levels of the pathway [50, 51] (Fig. 2). Briefly, TRP is metabolised by TDO, IDO1 or IDO2 into kynurenine (KYN). KYN is a central metabolite in the KP. It can be further metabolised by three different enzymes: kynurenine aminotransferase (KATs), kynureninase (KYNU) or kynurenine monooxygenase (KMO) to produce the intermediates kynurenic acid (KYNA), anthranilic acid (AA) and 3-hydroxykynurenine (3HK), respectively (Fig. 2).

The preferred catabolic route for KYN in the brain is to produce KYNA by KATs under normal physiological condition [52]. Due to the low affinity of KYN to KYNU, the conversion of KYN to AA only occurs when the concentration of KYN is considerably elevated [53]. The oxidation-reduction converts AA further to 3-hydroxyanthranilic acid (3HAA) [54]. The KMO route is more active in the peripheral tissues, particularly in the liver and kidney, and less active in the brain [55, 56]. The metabolite 3HK can then be subsequently converted to 3HAA by KYNU, to xanthurenic acid (XA) by KATs or even to 3-hydroxy-L-kynurenamine (3HKA) [57]. The metabolite 3HAA can give rise to cinnabarinic acid (CA) by spontaneous auto-oxidation [58] or to 2-amino-3-carboxymuconate semialdehyde (ACMS) by the enzyme 3-hydroxyanthranilate dioxygenase (HAAO). Next, ACMS can either non-enzymatically form quinolinic acid (QUIN), a vital precursor for de novo synthesis of NAD^+^ or picolinic acid (PIC) by 2-amino-3-carboxymuconate semialdehyde decarboxylase (ACMSD) [59].

As mentioned above, the KP has a major role in physiological processes through the supply of NAD^+^, a co-factor required in many biochemical processes. In some mammals, the KP can supply sufficient NAD^+^ in the absence of any dietary niacin intake [60]. Another established biological role for the KP is the regulation of the immune response [61]. It has been suggested to be involved in the maintenance of immunological tolerance and immune privilege in the eyes, brain, placenta, hair follicles and colon [62, 63].

Initially, the KP was simply thought to function as an immunomodulatory mechanism by allowing cells to deplete TRP from the intracellular pool or local microenvironment and, therefore, lead to the suppression of T-cell function and growth [64]. Accumulating evidence has demonstrated that KP-mediated immunoregulatory processes are also triggered by the biological activity of KP metabolites. Several KP metabolites are ligands of the aryl hydrocarbon receptor (AhR), a ligand-operated transcription factor. They have been shown to modify the behaviour of immune cells through a more tolerogenic phenotype [65, 66]. Several recent studies have suggested a link between the KP-dependent activation of AhR and the development and normal functioning of immune systems [65–68]. For instance, KP-mediated activation of AhR in intestinal epithelia is essential for developing and maintaining intestinal barrier function, a mechanism that, when disrupted, can contribute to inflammatory bowel diseases [69].

### Roles of the KP in cancer

**Fig. 3:**
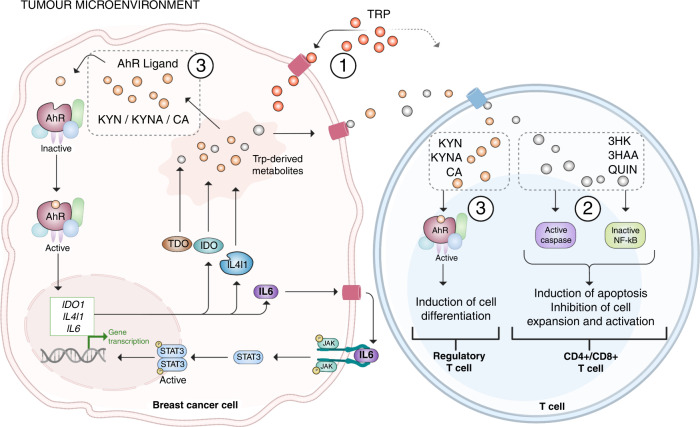
The kynurenine pathway (KP) capacity to suppress local immune surveillance and favour tumour growth. Three mechanisms are proposed for the suppression of local immune surveillance by the KP: (1) An overactive tumoral IDO1/ TDO can reduce the TRP availability in the tumour microenvironment and ultimately lead to T-cell proliferation arrest and increased ability of cancer cells to escape T-cell-dependent antitumor immunity. (2) KP metabolites 3HK, 3HAA and QUIN can suppress anti-tumour immunity. 3HK, 3HAA and QUIN induce apoptosis of anti-tumour immune cell populations such as CD4^+^ and CD8^+^ T via caspase activation, while 3HAA can induce CD4^+^ T-cell death via NF-κB inhibition. (3) The KP metabolites KYN, KYNA and CA are AhR ligands. Activation of the AhR on T cells reduces T-cell differentiation and activation. Activation of the AhR on the tumour cells induces an inflammatory positive autocrine feedback loop that enhance the production of AhR ligand and immunosuppressive KP metabolites.

The involvement of the KP in cancer was first observed in early studies where cancer patients were reported to have higher KP activity [70, 71]. As compared to healthy controls, cancer patients were shown to have lower concentrations of TRP and higher concentrations of KP metabolites in their blood and urine. The pivotal study that provides the most substantial evidence of the KP involvement in cancer is the study by Muller et al. They demonstrated that combination therapy of a chemotherapeutic drug and an IDO1 inhibitor, 1-methyl-D-tryptophan (1MT), reduced the tumour size by 30% in a HER2-enriched mouse model. Furthermore, this treatment is highly dependent on a competent immune system [72]. Since the outcome of this study, the KP has emerged as a potential target to address cancer progression and metastasis [73]. The attention on KP and cancer is centred on its capacity to suppress local immune surveillance, and there are at least three different mechanisms (Fig. 3). As TRP is one of the critical components required for T-cell survival, a first mechanism involves the overactivation of IDO1 and/or TDO to deplete TRP within the local tumour microenvironment rapidly. Consequently, a TRP-stripped tumour microenvironment mediated by an overactive tumoral IDO1/TDO will induce mid-G1 phase arrest in T cells [74]. Elevated IDO1/TDO activity has been reported in tumour cells and blood sampled from patients with cancer, including glioblastoma [75, 76], colorectal cancer [77, 78], breast cancer [79, 80] and liver cancer [81]. This notion was supported by evidence of high IDO1 expression detected at metastatic sites and that the expression of IDO1 correlates positively with the immune-suppressive T regulatory population [82–85]. The association between the KP and various cancers indicates that the KP may play an important role in cancer progression.

The second immune suppression mechanism of the KP involves its metabolites and their interaction with immune T cells and tumours (Fig. 3). Notably, three KP metabolites 3HK, 3HAA and QUIN were shown to suppress anti-tumour immune cell populations. Of these KP metabolites, 3HAA has the most potent immune-suppressive capability because of the number of T-cell populations it interacts with. In vitro studies showed that 3HAA selectively induces apoptosis in CD4^+^ T-cell populations through the caspase activation pathway while inhibiting CD8^+^ T-cell expansion and activation [86, 87]. Additionally, 3HAA is capable of skewing T-cell differentiation towards regulatory T cells instead of anti-tumour T cells [88]. On the other hand, 3HK and QUIN were shown to induce cell death in CD4^+^ T cells [86, 89]. Apart from its immune-suppressive properties, QUIN was also shown to enhance glioblastoma cell survival when in excess. The enhanced cell survival may be due to the excess QUIN increasing the availability of NAD^+^ when catabolised. This NAD^+^ is then utilised in DNA repair, essentially reducing the apoptosis that was triggered by chemotherapeutic drugs [90].

A last immune-suppressive mechanism of the KP is mediated through the interaction between some KP metabolites and the ligand-activated transcription factor, the AhR. The AhR is expressed in all cell types, and its activation can trigger various biological pathways regulating the immune response and various cellular functions [91]. Other than KYN, KYNA, and cinnabarinic acid (CA), the following metabolites indole-3-pyruvic acid (I3P), indole-3-acetic acid, and indole-3-carboxaldehyde are also known ligands to the AhR [92]. Interestingly, the type of biological pathway activated depends on the cell type on which AhR is activated. T-cell differentiation and activation is reduced when AhR is activated on the T cells by KYN [76]. Activation of AhR on the tumour by KYN induces an inflammatory positive autocrine feedback loop (IDO1-AhR-IL-6-STAT3 signalling pathway) to enhance tumour growth [93]. This pro-inflammatory feedback loop can also be triggered by KYNA [94].

The KYNA triggered observed feedback loop is produced by the activation of the Interleukin-4 induced gene 1 (IL4I1) rather than the KP. Sadik et al. showed that melanoma has high expression of IL4I1 producing KYNA, and KYNA activation of the AhR enhanced tumour cell motility and T-cell proliferation [95]. Elevated of IL4I1 activity is not affected by IDO1 inhibition, suggesting that this new mechanism may be leading to IDO1 and/or immune checkpoint inhibition resistance.

### Involvement of the KP in BrCa

We conducted a search on PubMed using the keywords “kynurenine” and “breast cancer” and we found 58 studies that looked at the KP in BrCa. These studies and their outcomes are summarised in Table 1.

**Table 1:** Summary of all KP research carried out on BrCa.

	Year	Sample	Assay	KP target	Outcome	Reference
1	1967	Patient cohort: 20 BrCa patients treated by mastectomy alone, 24 BrCa patients treated with oophorectomy and mastectomy and 15 healthy controls	Thin-layer chromatography and column chromatography	Metabolite: XA, 3HK and 3HAA	• 13 patients who received mastectomy only had more KP metabolites while 12 patients who received combination treatment had less KP metabolites as compared to control. • 8 patients who received combination treatment had increased excretion of the KP metabolites due to increased adrenal production of oestrogen after oophorectomy.	[96]
2	1972	Patient cohort: 32 healthy control, 36 early BrCa, 9 BrCa patients with bone metastasis, 15 BrCa patients with soft tissue metastasis, 16 patients with cervical cancer	Ion-exchange chromatography, thin-layer chromatography	Metabolite: XA, 3HK, 3HAA	Increased detection of KP metabolites in patients with early BrCa and soft tissue metastases cohort but not in bone metastasis cohort.	[99]
3	1973	Patient cohort: 5 BrCa patients with previous mastectomy and no metastasis, 2 with local recurrence, 7 with ossesous metastases, 11 with visceral metastases; 12 healthy women as controls.	No data access	Metabolite: 3HK, KYN, KYNA, XA and AA	• There was no difference in the basal urinary KP metabolites level (3HK, KYN, KYNA, XA and AA) between BrCa patients and controls. • Half of the BrCa cohort had higher concentration of KYN, AA, XA and KYNA detected in their urine after TRP loading.	[98]
4	1978	19 British BrCa women and 20 Japanese BrCa women	Protein binding assay	Metabolite: KYN, XA, 3HK and 3HAA	• No difference was detected in the level of KYN, 3HK, 3HAA and XA between the two races. A correlation with plasma estradiol, KYN and other metabolites was observed in British women but not in Japanese women.	[97]
5	2004	Cell line: human BrCa cells (MCF-7 and MDA-MB-231)	qPCR, HPLC	Enzyme: IDO1 Metabolite: TRP, KYN	• MDA-MB-231 had expression and functional IDO1 but not MCF-7. The IDO1 activity was inhibited by 1-MT. Transport of TRP is through the LAT1 (BCH-sensitive, Na + -independent pathway) in MDA-MB-231	[128]
6	2005	Animal: (NMU and 13762) Cell line: human BrCa cells (MCF-7 and T47D)	Immunohistochemistry (IHC), western blot, Enzymatic assay, flow cytometry	Enzyme: IDO1	• Haem-oxygenase-1 (HO-1) overexpression inhibited BrCa growth and induced proapoptosis by antioxidant mechanism in all 4 cell lines. • HO-1 negative human BrCa cell lines showed increased proliferation after IDO1 inhibition. • HO-1 inhibited IDO1 at post-translational level by starvation of haem. Normal rat mammary tissue showed co-expression of HO-1 and IDO1 while cancer tissue only showed IDO1.	[129]
7	2008	Patient cohort: BrCa patients with recurrent cancer receiving chemotherapy (no access to cohort size)	HPLC	Metabolite: TRP, KYN	There was no difference in IDO1 activity between pre- and post-paclitaxel treated BrCa patients. However, IDO1 activity was higher in patients treated with docetaxel therapy as compared to pre-treatment.	[135]
8	2009	Patient cohort: 30 patients with malignant BrCa and 27 patients with benign breast disease	HPLC, ELISA	Metabolite: TRP, KYN	Patients with higher grade of BrCa had higher TRP degradation which was correlated with elevated neopterin concentration in serum.	[168]
9	2011	Patient cohort: Recently diagnosed BrCa patients who are being treated with chemotherapy and healthy controls (no information on cohort size)	HPLC	Metabolite: TRP, KYN	• BrCa patients had higher K/T ratio as compared to healthy controls • K/T ratio decreased after last cycle of chemotherapy	[108]
10	2011	Patient cohort: Two cohort pre- and post-chemotherapy treatment: (1) Patients receiving chemotherapy and (2) patients receiving hormone therapy (no access to cohort size)	HPLC	Metabolite: TRP, KYN	• Patients who received chemotherapy had higher IDO1 activity after treatment with chemotherapy. • There was no significant difference in IDO1 activity from pre- and post- hormone therapy.	[133]
11	2012	Patient cohort: 9 women with a diagnosis of BrCa. 8 women had a newly diagnosed tumour and 1 woman had a history of BrCa.	Position emission tomography and IHC	Enzyme: IDO1	• Moderate to high expression of L-type amino acid transporter 1, IDO and tryptophan hydroxylase in most tumour cells.	[109]
12	2012	Patient cohort: BrCa patients with bone metastases (no access to cohort size)	HPLC	Metabolite: TRP, KYN	• Activity of IDO increases in patients with higher number of metastatic lesions to the bone.	[105]
13	2012	Patient cohort: Three cohorts: (1) patients receiving chemotherapy only, (2) patients receiving tratuzumab + chemotherapy and (3) patients receiving hormone therapy + chemotherapy. Two time points: pre- and post-chemotherapies.	HPLC	Metabolite: TRP, KYN	• IDO1 activity is significantly lower post-chemotherapy compared to post hormone therapy. • Comparing between two cohorts in post-therapy phase, patients who have received chemotherapy have the lowest IDO1 activity. • Patients who received trastuzumab showed no difference pre and post-therapy suggesting that this treatment is less invasive than chemotherapy.	[119]
14	2013	Patient cohort: 32 BrCa patients who had cancer recurred 5 or more years after surgery; two cohorts: (1) 12 patients were selected for locoregional therapy (2) 20 patients selected for chemotherapy	HPLC	Metabolite: TRP, KYN	Patients receiving locoregional therapy had higher IDO1 activity as compared to chemotherapy cohort and were associated with better prognosis.	[132]
15	2013	Patient cohort: BrCa patients treated with neoadjuvant chemotherapy; three time points: pre- and post-chemotherapy and post-surgery chemotherapy treatment (no access to cohort size)	HPLC	Metabolite: TRP, KYN	IDO1 activity is lower in patients after receiving chemotherapy. However, there is no significant changes between the time points.	[118]
16	2013	(No access to cohort size)	HPLC, ELISA, Meso scale discovery multi-spot assay (cytokines)	Metabolite: TRP, KYN	• High KYN concentration is associated with increased immune activation but has no impact on fatigue or depression. • KYN concentration in blood pre- and post-treatment showed significant potential to predict changes in depression.	[169]
17	2014	Patient cohort: 25 BrCa (23 fully analysed by TCGA) and 5 normal breast specimens	Gas Chromatography/Mass Spectrometry (GC/MS) and Liquid Chromatography/Mass Spectrometry (LC/MS)	Enzyme: IDO1 Metabolite: TRP, KYN	Expression of IDO1 correlates with level of KYN and basal phenotype as compared to ER- cancers	[111]
18	2014	Cell line: MCF-7 and MDA-MB-231	ELISA, shRNA,	Enzyme: IDO1 Metabolite: TRP, KYN	• Level of KYN was elevated in co-culture of fibroblasts and COX-2-overexpressing BrCa cells. • PGE2 released by BrCa cells upregulates IDO expression in fibroblasts	[112]
19	2014	Cell line: 122 tissue and serum samples were used (91 malignant, 21 benign, and 10 normal). MCF7, MDA-MB-231 and T47D.	IHC, immunocytochemistry (ICC), western blot and RT-PCR	Enzyme: IDO1	• Data indicated that the expression of IDO1 was higher in BrCa and increased with higher stages • IDO was mainly expressed in the TN BrCa subtype	[115]
20	2014	Patient cohort: 11 BrCa patients with local recurrence and 26 BrCa with distant metastases.	HPLC	Metabolite: TRP, KYN	• IDO1 activity was observed to be higher in patients with local recurrence. This was also observed in patients with single metastatic lesion. • IDO1 activity was noted to decline when patients were receiving chemotherapy.	[106]
21	2015	Cell line: MCF7, MCF10AT1, MDA-MB-231 and SK-BR3	Apoptosis assay, siRNA, qPCR, western blot	Enzyme: IDO1	• Activation of AhR by TCDD suppresses apoptotic response induced by UV-irradiation and chemotherapy drugs • TCDD induced inflammatory genes such as cyclooxygensase-2 and NF-kB subunit RelB TCDD also induced IDO1 to produce KYN; this production mediated an anti-apoptotic response in BrCa cells.	[140]
22	2015	Patient cohort: 51 mentally healthy patients with BrCa, 29 patients with a diagnosis of depression and BrCa, 46 patients with depression without BrCa and 28 mentally and physically health patients as matched controls.	HPLC	Metabolite: TRP, KYN	• Significant increase of Kyn/Trp ratio was seen in BrCa patients • There was no significant effect of depression on the Kyn/Trp ratio.	[107]
23	2015	Patient cohort: 69 formalin-fixed and paraffin-embedded BrCa tissue microarray	IHC	Enzyme: IDO1 Metabolite: KYN	• 18 of 69 samples had detectable KYN staining in the cytoplasm of the tumour	[170]
24	2015	Cell line: MDA_MB_231, MCF7, T47D, SUM159PT, BT549	HPLC, qPCR, western Blot, IHC, gene-expression array analysis, shRNA, Incucyte ZOOM live-cell imaging system	Enzyme: TDO, KYNU	• KP was elevated in non-adhered or suspension BrCa cells • KYN produced by TDO2 activates AhR • Pharmacologic inhibition or genetic attenuation of either TDO2 or AhR reduced cell proliferation, migration, and invasion of TNBC while increasing sensitivity to anoikis • NF-kB regulates TDO2, KYNU and AhR in forced suspension TNBC	[82]
25	2016	Patient cohort: TCGA dataset (*n* = 977)	IHC, qPCR, western blot, LC/MS, colorimetric KYN assay, shRNA, CRISPR-Cas9, Scratch-wound assay	Enzyme: TDO Metabolite: KYN, XA, KYNA	• TCGA dataset showed that primary BrCa and its metastatic lesions express high levels of AhR and TDO. • TNBC expressed TDO and produced sufficient KYN and XA to activate AhR • AhR regulated TDO to elevate production of XA and KYN Activation of AhR by KP metabolites enhanced tumour cell migration.	[138]
26	2016	Patient cohort: BrCa patients with multiple metastatic lesions were treated with toremifene fulvestrant. (no access to cohort size)	HPLC	Metabolite: TRP, KYN	• IDO1 activity was lower in patients with multiple metastatic lesions than patients without metastases. • IDO1 activity increased when BrCa metastases developed and IDO1 activity was correlated with the number of metastatic lesions during tormefine and fulvestrant treatment.	[117]
27	2017	Patient cohort: 32 healthy control, 36 early BrCa, 9 BrCa patients with bone metastasis, 15 BrCa patients with soft tissue metastasis, 16 patients with cervical cancer	HPLC	Metabolite: TRP, KYN	• BrCa patients with later stage had lower KP ratio than other stages. • BrCa patients over the age of 70 has lower K/T ratio than those lower than age of 70. • Within each stage, patients over age of 70 has lower K/T ratio those below age of 70.	[171]
28	2017	Animal: Female SCID mice Cell line: MDA-MB-231	micro-PET/CT imaging,	Enzyme: IDO1	• 1-L-[18 F]FETrp is a promising radiotracer for PET imaging of IDO1 activity in animal model	[172]
29	2017	Patient cohort: Tissue microarray of 362 patients diagnosed with stage I–III BrCa	IF	Enzyme: IDO1	• IDO1 protein was detected in 76.2% of hormone receptor positive BrCa • IDO1 expression was not associated with any clinical characteristics, CD3 + , CD8 + or FOXP3. • Increased IDO1 expression was associated with decreased CD20 +	[103]
30	2017	Patient cohort: sera from 46 BrCa patients (30 from untreated ER + patients and 16 untreated ER- patients) 6 ER- and 9 ER + BrCa tissue cryosamples Cell line: MCF7, BT-474, ZR-75-1 and HCC1954 (colorectal cancer)	IHC, western Blot, HPLC, MassARRAY, qPCR, ELISA	Enzyme: IDO1 Metabolite: TRP, KYN	• ER is a negative regulator of IDO1 expression • Serum level of KYN and IDO1 expression in tumour was lower in ER + tumours as compared to ER- tumours • IDO1 promoter hypermethylated in ER + as compared to ER- BrCa • Hypermethylation of IDO1 promoter is unique to ER + BrCa and not observed in cervical or endometrial cancer	[104]
31	2017	Patient cohort: Various time points in 3 patients with locally advanced BrCa receiving Mohs paste treatment	HPLC	Metabolite: TRP, KYN	• There is no difference in IDO1 activity during Mohs treatment, thus suggesting that Mohs paste treatment does not induce KP-mediated immunosuppression.	[134]
32	2017	Patient cohort: Patient treated with Letrozole therapy (no access to patient number)	HPLC		• IDO1 activity decreased after receiving Letrozole and was correlated to the number of metastatic lymph node lesions.	[121]
33	2018	Patient cohort: Elderly patients with oestrogen receptor positive BrCa and not receiving surgery or chemotherapy due to old age. (no access to cohort size)	HPLC	Metabolite: TRP, KYN	• IDO1 activity and tumour size decreased after letrozole therapy.	[120]
34	2018	Patient cohort: 439 BrCa patients treated with neoadjuvant chemotherapy; three time points: pre- and post-chemotherapy and post-surgery chemotherapy treatment Cell line: MDA-MB-436	qPCR and IHC	Enzyme: IDO1, TDO	• A weak association between high AhR expression and age > 50 years old and oestrogen • AhR repressor mRNA but not AhR mRNA was associated with metastasis-free survival • high AhR expression was associated with high expression of IDO and TDO gene expression.	[139]
35	2018	Cell line: BT549, Hs578T, SUM159 and MDA-MB-453	uHPLC-MS, qPCR, western blot, flow cytometry, gene-expression analysis, soft-agar assays	Enzyme: TDO	• miR-200c suppresses genes involved in epithelial-to-mesenchymal transition and is in low abundance in TNBC • miRNA-200c suppresses TDO2 protein expression and its activity.	[137]
36	2018	Patient cohort: 19 patients with early-stage BrCa	LC/MS	Metabolite: TRP, KYN	• Significant increase in KYN and K/T ratio in patients’ post-chemotherapy • Symptoms of pain and fatigue showed strong associations with KP metabolite and K/T ratio.	[131]
37	2018	Patient cohort: 65 paraffin sections from BrCa patients Cell line: MDA-MB-231, MDA-MB-435S, MDA-MB-453, SK-BR3, ZR-75-1, T47D and MCF_7 Primary cell: Human umbilical vein endothelial cells	qPCR, IHC, HPLC	Enzyme: IDO1	• IDO1 expression showed a positive correlation with microvessel density in tumour tissues and these correlations were associated with the clinical profile of BrCa • Patients with IDO protein expression or microvessel density were associated with shorter overall survival as compared to non-IDO expression or/and microvessel density levels. • IDO mRNA expression was detected in all BrCa cell lines • MCF-7 enhanced the growth of human umbilical vein endothelial cells when co-cultured. This cancer mediated growth enhancement effect was inhibited by addition of 1-MT	[113]
38	2018	Patient cohort: 60 stage I/II BrCa patients who underwent surgery	Magnetic Resonance spectroscopy (MRS), MS	Metabolite: TRP, KYN	• KYN levels were increased 6 months after surgery in both patients receiving and not receiving chemotherapy (38% and 25% increases respectively)- only significant in chemotherapy patients.	[173]
39	2018	Animal: BALB/c mice Cell line: 4T1 murine cancer cell line	HLPC, LC/MS, Annexin-V, ELISA and IHC	Metabolite: KYN	• PEG-KYNase is a recombinant kynureninase that degrades KYN. Treatment of these tumours in combination therapy (PEG-KYNase and anti-CTLA4) showed a 45% increase in median survival.	[174]
40	2019	Patient cohort: 96 BrCa patients divided into non exercising control group (CG) and exercising group intervention group (IG). 24 healthy women were included in IG.	ELISA, ANCOVA (statistical analysis)	Metabolite: KYN, QA AND KYNA	• Serum KYN levels and the KYN/TRP ratio were significantly reduced in intervention group compared to control group both at timepoint 1 and timepoint 2. • Comparison of the effects of resistance exercise between BrCa patients (IG) and healthy women (IG) revealed that while urine QA levels and the QA/KYNA ratio were reduced in the IG healthy subjects, IG BrCa patients showed reduced KYNA/KYN ratios and elevated QUINA/KYNA ratios after completion of the 12-week exercise training	[175]
41	2019	Patient cohort: 77 BrCa patients and 40 healthy controls Cell line: BT549	UHPLC-MS, flow cytometry	Enzyme: IDO1 Metabolite: TRP, KYN	• KYN induces cell death in CD8 T cells through activation of AhR • Expression of TDO is associated with poor clinical outcome (overall survival and distant metastasis-free survival) but not IDO1 • BrCa patients have lower KYN and TRP as compared to healthy controls	[136]
42	2019	Patient cohort: 202 women with stages I–III BrCa and 146 healthy controls	LC-MS/MS	Enzyme: IDO1 Metabolite: TRP, KYN	• Healthy controls have higher level of KYN, TRP and K/T ratios as compared to BrCa patients • Patients with ER- BrCa had higher K/T ratios than patients with ER + BrCa • Within the cancer patients, those with advanced stage cancer were associated with higher K/T ratios	[114]
43	2019	Animal: Female BALB/c mice Cell line: 4T1 breast cancer cells	Flow cytometric analysis, IHC, shRNA (IDO1), ELISA and western blot	Enzyme: IDO1 Metabolite: TRP, KYN	• Doxorubicin treatment induced cell death in 4T1 cancer cells while NLG919 (IDO1 inhibitor) blocked KYN production and reversed CD8 T-cell suppression • Combination treatment significantly inhibited tumour growth, increased transforming growth factor-B while lowering IL12p70 and IFN-Y	[122]
44	2020	Animal: BALB/c mice and female ICR mice Cell line: 4T1 breast cancer cells, A549 (human lung cancer cell line), HepG2 (human liver cancer cell line)	Confocal laser scanning microscopy, flow cytometry, MTT assay, western blot, uHPLC-MS/MS, ELISA, IF, transferase-mediated deoxyuridine triphosphate-biotin nick end labelling assay,	Enzyme: IDO1 Metabolite: TRP, KYN	• Dual-functional prodrug that delivered simultaneous 1-MT and doxorubicin inhibited cell proliferation and induced apoptosis in cancer cells • Inhibition of IDO1 enhanced the population of CD4 and CD8 T cells while reducing Tregs population • Prodrug prolonged 4T1 mouse model	[126]
45	2020	Cell line: MDA-MB 231, MCF-7 cells, HeLa cells (cervical cancer) and HEK293A cells (human embryonic kidney cells) Animal: BLAB/c mice with 4T1 murine cancer cells	HPLC, IDO1/TDO cellular assay, IDO activity assay, western Blot, qPCR, CCK-8 assay, T-cell proliferation assay, IHC	Enzyme: IDO1 Metabolite: TRP, KYN	• Salinomycin has shown an inhibitory effect on the protein expression and activity of IDO1 • Inhibitory mechanism was induced through interaction with JAK/STAT and NF-kB pathways • Salinomycin enhances proliferation of T cells and effect of cisplatin in vivo	[125]
46	2020	Patient cohort: 53 BrCa patients Cell line: MCF-7, MDA-MB-231 and SKBR-3	IHC, HPLC, qPCR	Enzyme: IDO1	• IDO expression in both tumour and serum were associated with clinical tumour stage, node stage and ER status	[110]
47	2020	Patient cohort: 82 matched BrCa tumour and adjacent non-cancerous tissues	qPCR	Enzyme: IDO1 and TDO	• No significant difference in IDO1 expression between malignant tissues and adjacent non-cancerous tissues. • MicroRNA *HCP5* was lower while *ITGB2-AS1* was higher in in tumour tissue as compared to adjacent non-cancerous tissues. • Gene expression of TDO2 expression levels correlates to the expression of IDO1 and all microRNA genes in adjacent non-cancerous tissues	[116]
48	2020	Animal: 4T1 mouse model Drug: Nanoparticle (immuno-nanoagent; CaIPC)	MTT assay, transmission electron microscopy, flow cytometry	Enzyme: IDO1	• The immune-nanoagent induces dendritic cell maturation and activation of T cells • Co-stimulation of IDO1 inhibitor and release of calcium promotes activation and proliferation of T cells, leading to suppressed tumour progression and protects the mice from tumour re-challenge	[124]
49	2020	Patient cohort: 96 BrCa patients, Cell line: MCF10A cells	LC/MS, ELISA, IHC, MTT, colony-forming, transwell migration/invasion, and mammosphere assays, cDNA microarrays, western blot, qPCR	Enzyme: KMO	• KMO is amplified in TNBC and is associated with worse survival in BrCa patients. • KMO expression is elevated in TNBC tumours when compared to adjacent normal mammary tissues.	[141]
50	2020	Cell line: BT-20, T47D, SKBR-3, MCF-7, MDA-MB-468, MDA-MB-157, BT-474, DU4475 and MDA-MB-231 Animal: MMTV-PyVTmouse model	IHC, migration and invasion assay, immunoblot, RNA sequencing analysis	Enzyme: QPRT	• QPRT is upregulated in invasive and spontaneous mammary tumours. • Inhibition of QPRT reduces tumour invasiveness.	[176]
51	2020	Patient cohort: Cohort 1 (serum samples: 138 luminal, 127 HER2-enriched, and 143 TNBC and 98 healthy controls), Cohort 2 (formalin-fixed tumour tissues from 10 luminal, 10 HER2-enriched and 10 TNBC), Cohort 3 (Gene-expression values of 1140 luminal, 220 HER2-enriched, 199 TNBC and 144 healthy control). Cell line: MCF7, T47D, MBA-MB-231, MBA-MB-157, MBA-MD-468, HBL100, SK-BR3	qPCR, western blot, IHC and uHPLC	Enzyme: IDO1, KMO, KYNU Metabolite: TRP, KYN, 3HK, 3HAA, AA	• KP is elevated in HER2-enriched and TNBC subtypes, particularly KMO and KYNU, leading to increased production of AA and 3HAA.	[79]
52	2020	Cell line: MDA-MB–231	Voltametric determinations, HPLC	Metabolite: TRP, KYN	• The results obtained by both methods were in good agreement, confirming acceptable sensor accuracy in cell culture medium matrix. The voltametric method is preferred to the chromatographic run as it has a shorter processing time when monitoring TRP and KYN in human cancer cell lines.	[177]
53	2021	Cell line: MCF7	Western blot, mammosphere formation assay, MTS assay, Luciferase Reporter Gene Assay	Metabolite: TRP, KYN	• KYN showed strong induction of the transcriptional activation of AHR after 6 h, but its activation was reduced at 24 h • KYN-activated AHR does not suppress mammosphere formation of cancer stem cells isolated from MCF7	[178]
54	2021	Patient cohort: cohort 1 (monotherapy) 21 patients; cohort 2 (combination therapy) 21 patients (14 were TNBC).	LC-MS/MS	Enzyme: IDO1 Metabolite: TRP, KYN	• Combination treatment of LY3381916 (IDO1 inhibitor) with LY3300054 (PD-L1 inhibitor) showed maximal inhibition of IDO1 activity in plasma and tumour tissue, and increased CD8 + T cells within tumour tissue • Limited clinical activity observed in combination treatment.	[127]
55	2021	Patient cohort: 107 BrCa patients	HPLC, Hospital Anxiety and Depression Scale (HADS)	Metabolite: KYN	• High KYN levels post BrCa predicted both anxiety and depression	[179]
56	2021	Patient cohort: Cohort 1: 958 BrCa patients (TCGA), 55 BrCa patients (University of California, Santa Cruz). Cohort 2: Tissue microarray of 9 normal breast tissues and 9 matched BrCa tissues. Cell lines: MCF10A, MDA-MB-468, MDA-MB-453, HCC1954, MCF7, HCC1937, MDA-MB-361 and Hs578T Animal: Male BALB/c mouse model	Immunofluorescence (IF), flow cytometry, IHC of TMA, transmission electron microscopy, cytotoxicity assay, migration and invasion assay	Enzyme: KMO	• KMO amplification is higher in invasive BrCa than in 27 other cancers • KMO gene alterations were related to survival outcomes • KMO is overexpressed on the cell membranes of BrCa cell lines	[142]
57	2021	Patient cohort: 360 BrCa tumour tissues and 88 paired normal tissues	IHC, IF, Human Tregs and co-culture system, flow cytometry, qPCR, western Blot	Enzyme: IDO1 Metabolite: KYN	• GCH1 overexpression upregulates IDO1 • IDO1 is upregulated in TNBC	[102]
58	2021	Patient cohort: Oncomine Database (715 datasets of 86, 733 cancer samples), Genotype-Tissue Expression and Cancer Genome Atlas database (9736 tumours and 8587 normal samples)	Gene Expression Profiling Interactive Analysis, cBioportal DataBase analysis, University of California Santa Cruz Cancer Genomics Analysis, Breast Cancer Gene-Expression Miner v4.4	Enzyme: KMO	• KMO was overexpressed in invasive ductal BrCa • Expression of KMO showed positive correlation with malignant clinical characteristics of BrCa patients • Top ten KMO correlated genes were pro-inflammatory cytokines and chemokines	[143]

The possibility that TRP metabolism may have a role in BrCa was alluded to in early studies from the 1970’s by measurement of 3HAA, 3HK, AA, KYN, KYNA and XA in the urine of patients following ingestion of TRP [96–99]. Two studies showed that ~50% of patients with BrCa have higher rates of KP metabolism as compared to controls [96, 98]. This skewed observation of BrCa patients with elevated KP metabolism could be due to the distribution of BrCa subtypes in their study cohort [79] and not attributed to the ethnicity of the patients as this study showed no difference in KP metabolism between two ethnic groups [97]. A later study reported that patients with soft tissue metastases have a higher level of KP metabolism as compared to patients with bone metastases due to higher concentration of 3HK, XA and 3HAA in their urine samples [99]. Despite these findings from the 1970’s, no further studies were conducted until the early 2000’s, when research delved deeper into the regulation of the KP in BrCa and the potential role of KP metabolites and enzymes in the pathology of BrCa.

The advancements in analytical chemistry and molecular biology techniques may have led to an increased number of studies on the KP in BrCa after the year 2000. In particular, analytical chemistry techniques such as high-performance liquid chromatography (HPLC) have provided high-resolution separation and quantitation of components from a biological matrix with greater accuracy, sensitivity and reproducibility of results, which has made it an appropriate method to quantify chemical constituents in biological samples [100]. HPLC has emerged as a key technique used in the majority, if not all of the studies conducted after 2000 that explore the impact of IDO1 in BrCa by direct assessment of the pathway activity using the blood of patients or cell cultures. The common approach to measure IDO1 activity is by quantifying the primary substrate and immediate product of IDO1 enzymatic reaction. The activity of the enzyme can be calculated by a ratio of product and substrate and can be readily measured now from blood using simple ELISA kits (ImmuSmol). Blood collection is routinely performed, safe and inexpensive. However, the quantification of downstream KP metabolites require specialised and often large and expensive analytic equipment. Another limitation of this quantification methodology is that the ratio is a systemic measurement instead of a snapshot of the tumour environment. The KYN/TRP ratio is not specific only to IDO1 but also includes the activity of TDO (Fig. 2). These limitations can be addressed by quantifying the expression of IDO1 enzyme in tumour tissue using immunohistochemistry and/or the flow cytometry. The KYN/TRP ratio is the preferred approach as the latter techniques are usually time consuming and expensive. Both techniques also require surgery and the removal of tumour tissue from patients, which is more invasive when compared to blood collection. While this provides accurate levels and cellular localisation of IDO1 expression in the tissues, it does not provide information if this enzyme activity is inhibited or not. Hence, studies usually combine both techniques to provide an accurate overview of IDO1 activity in the tumour and to assess if its activity is inhibited when an inhibitor is applied.

Since the early 2000’s, there were 40 studies examining the role of IDO1 in BrCa that includes the impact of chemotherapy on IDO1 activity and inhibition of IDO1 itself. As noted above, IDO1 is a significant rate-limiting enzyme that regulates the production of KP metabolites and is generally highly expressed in multiple human cancers [101]. Although most of the studies reported a positive correlation between BrCa progression and activity of IDO1 [102–115], one study showed the opposite observation [114] while another reported no significant differences in the expression of IDO1 between cancerous and non-cancerous tissues [116]. Inconsistent observations on the activity of IDO1 was also reported in patients with metastatic BrCa, even though these observations derived from studies that were conducted by the same research group. Their early study reported that expression of IDO1 positively correlates with the number of metastatic lesions [105] while their later studies showed the IDO1 activity was lower in patients with high number of metastatic lesions as compared to patients with no or lower number of metastatic lesions [106, 117]. The observed discrepancies could be due to the distribution of BrCa subtypes in their cohort as the KP was found to be highly elevated in HER2-enriched and TNBC but not luminal [79].

Interestingly, chemotherapy treatment reduced the activity of IDO1 in serum samples of BrCa patients and animal model study [106, 108, 118–122]. This suggests that the IDO1 may play a role in either tumour growth or tumour environment in BrCa. Given the evidence that IDO1 promotes T-cell anergy and suppresses tumour control [123], studies were carried out to examine the potential of inhibiting IDO1 to reduce tumour growth to unravel its role in BrCa progression. Results from in vitro, in vivo and clinical studies have revealed that the inhibition of IDO1 led to tumour suppression [122, 124–129]. This suppression of tumour is associated with an increased production of T cells such as CD4 + and CD8 + cells as well as an increased apoptosis of cancer cells [126, 127]. Intriguingly, a study showed that the efficacy of IDO1 inhibition is dependent on the activity of haem-oxygenase-1 (HO-1) [129]. HO-1 is a rate-limiting enzyme that controls the haem catabolism [130] and inactivity of both enzymes was suggested to release all available haem for the cancer cells to proliferate.

The above studies support the notion that IDO1 is elevated in tumours to suppress immune surveillance and favour tumour growth, suggesting IDO1 as a potential therapeutic target to treat BrCa. It is also important to note that there were studies showing either an increase in IDO1 activity [131–133] or no difference in IDO1 activity [134, 135] after receiving chemotherapy such as paclitaxel, Mohs paste or surgery. While there is limited information on the patient cohort, potential explanations for these different observations could be due to proportion of the subtypes of BrCa and/or the percentage of patients with IDO1 overexpressing-tumours enroled in these studies. Considering that there are also other rate-limiting enzymes besides IDO1, recent studies have examined TDO, and the KP downstream enzyme KMO and their interactions with other pathways. Five studies have reported that TDO levels have a positive correlation with worse overall survival, increased disease grade and invasion/migration capability [136]. Additionally, the expression of TDO is often detected concomitantly with the expression of IDO1 [82, 116, 137–139]. Furthermore, the expression of TDO showed a strong correlation with AhR and has been associated with enhanced tumour cell migration [82, 138–140]. KMO was reported to be elevated in the TNBC subtype and was associated with worse survival and malignant characteristics such as node-positive status. Invasive BrCa has been shown to have the highest KMO amplification among all human cancers [79, 141–143].

### Clinical trials of IDO1 inhibitors in BrCa patients

IDO1 has emerged as an attractive pharmacological target as the enzyme has been well characterised and has high affinity for TRP among the small number of TRP catabolising enzymes (TDO, IDO2 and TPH). Given that it is the rate-limiting enzyme, inhibition of its activity may potentially block or reduce the activity of the whole KP, and its inhibitory action can be measured indirectly in the blood. As most of human cancers, including BrCa, overexpress IDO1, the validation of IDO1 inhibitor efficacy in enhancing anti-tumour immune activity could be translated for clinical use. With the positive outcome from the demonstrated efficacy of limiting tumour growth with combination treatment of IDO1 inhibition and chemotherapy drug(s) in in vivo and in vivo studies, 97 different clinical trials have been reported in ClinicalTrials.gov examining combination therapy in patients with cancers [144]. Among them are six clinical trials involving patients with BrCa; the various IDO1 inhibitors examined were epacadostat, indoximod and navoximod/GDC-0919 [144].

#### Epacadostat

Epacadostat has been shown to have the greatest efficacy in inhibiting IDO1 activity [145, 146] and in shifting the immune population towards tumour targeting CD8 + T cells [147]. The efficacy of epacadostat is based on preclinical studies that demonstrated it to have >100-fold selectivity for IDO1 as compared to other IDO1 inhibitors mentioned here. The administration of Epacadostat to tumour bearing syngeneic mice inhibited kynurenine production by ~90% in both tumour and plasma [146]. A solitary published trial, NCT02178722, is a phase I/II clinical trial that examined the efficacy of combining epacadostat with pembrolizumab to improve disease outcomes for patients with advanced solid tumours [148, 149]. In a phase I dosage escalation study, three of the 62 enroled patients were patients with TBNC, and only one achieved stable disease at the end of the phase. Within the cohort, tumour tissue from 22 participants were assessed for IDO1 expression. The assessment was carried out using the RNAscope technology and a scoring histoscore of more or equal to 5 was IDO1 positive. 13 patients were shown to have high IDO1 activity in tumour-infiltrating immune cells while 9 were evaluated to be negative as their histoscore was below the arbitrary cut-off value. The IDO1 status for the remaining patients remained unknown. Based on an encouraging clinical profile of more than 50% inhibition of IDO1 activity, 100 mg was chosen as the optimal dose for the Phase II study [148]. A total of 382 patients were enroled, with a selected schedule of 100 mg epacadostat with 200 mg of pembrolizumab. Thirty-six of the patient cohort were TNBC. Among the BrCa patients, one patient had a complete response, one showed very good response, two only minor response, while seven patients showed stable disease and 19 patients exhibited disease progression. Evaluation of disease progression could not be conducted in six patients. Overall, 51% of patients in the Phase II study experienced treatment-related adverse events; up to 15% of these adverse events were grade 3 or higher. At the conclusion of the study, TNBC was not considered for phase III inclusion [149]. NCT03328026, is a phase I/II trial currently recruiting patients to evaluate the combination treatment of SV-BR-1-GM with INCMGA00012 and epacadostat in metastatic or locally recurrent BrCa patients. This trial is currently recruiting and is expected to conclude on 31 December 2022.

#### Indoximod

Indoximod is the most studied IDO1 inhibitor in in vivo studies. It mimics TRP in reversing IDO1-mediated inhibition of the mammalian target of rapamycin complex 1 (mTORC1) [150, 151]. mTORC1 is one of the major signalling pathways regulating cell responses to intracellular nutrients such as TRP [152]. Two completed trials have examined Indoximod in the treatment of BrCa patients. NCT01792050 is a Phase II trial that observed the efficacy of a combination treatment of indoximod with taxane chemotherapy in patients with metastatic BrCa [153]. Recruitment criteria included HER2 negative tumours, normal organ function with the ability to receive taxane chemotherapy (paclitaxel or docetaxel), no history of autoimmune disease and no prior history of immunotherapy. A total of 169 patients were enroled in the trial. Results showed that there was no significant difference in the occurrence of toxic events and progression free survival between the combination therapy and single treatment arm (6.8 vs 9.5 months, respectively). Furthermore, there was no significative difference in response rates between the groups (40% and 37%) or overall survival (19.5 vs 20.6 months). Due to these lack of efficacy the study was discontinued prematurely. In determining its potential to be used as a predictor of response to indoximod, 52 archived tumour samples from the above reported study were stained for their IDO1 expression using IHC staining on these tumour samples. A cut-off score of 16.5 was derived as the median from previously stained samples. This score was used to classify the IDO1 expression as either high or low [153].

Although patients with high IDO1 expressing tumours (*n* = 30) were found to have longer progression free survival and overall survival than patients with low IDO1 expressing tumours (*n* = 22), the differences were not statistically significant. A later phase I/II trial, NCT01042535, examined the efficacy of combining indoximod with an adenovirus-p53 transduced dendritic cell vaccine in the treatment of patients with metastatic BrCa. Of the 39 patients enroled, 22 patients who obtained more than two cycles of post vaccination therapy, which included chemotherapy drugs, were evaluated for response. One patient achieved complete response, seven partial response and one remained with stable disease. Among these nine patients that continued into Phase II, five patients showed a 10% increase in activated CD8 + cells in their blood count. Despite a favourable outcome, this combination therapy did not achieve the pre-set threshold of a 20% objective response rate [154].

#### Navoximod/GDC-0919

Navoximod/GDC-0919 is an IDO1 inhibitor with selective inhibition against TDO [155–157]. Two clinical trials, NCT02048709 and NCT02471846, examined the potential of navoximod in the treatment of BrCa patients.

NCT02048709 is a Phase I trial that evaluated the safety profile of navoximod in patients with recurrent advanced solid tumours [158]. Out of the 22 patients enroled, only one patient was identified with BrCa and had received 28-day cycles of Navoximod 600 mg. The BrCa patient was later withdrawn from the trial based on the physician decision. This trial reported a dose dependent inhibition of IDO1 activity based on the concentration of plasma KYN. Navoximod was well tolerated at the maximal dosage of 800 mg BID or 600 mg on continuous dosing, and no maximal toxicity dosage was reached. However, this trial also reported that navoximod showed minimal anti-tumour activity.

NCT02471846 is a Phase I trial that investigated the safety and efficacy of navoximod in combination with the PD-L1 inhibitor, atezolizumab, in patients with locally advanced or metastatic solid tumours [159]. A total of 66 patients were enroled for the multiple ascending dose study of navoximod that included 13 patients with TNBC. The maximum toxicity dosage was not reached; the measurement of plasma KYN in these patients showed that the inhibition of IDO1 is dose dependent. However, none of the TNBC patients achieved any anti-tumour response. An expansion of the dose escalation cohort (dose expansion cohort) was conducted to recruit another 92 patients. Twelve enroled patients were TNBC. Only one patient from the total BrCa group achieved objective anti-tumour activity. A series of tumour biopsies was collected from the dose escalation and expansion cohorts pre- and on-treatment, and IHC of IDO1 was performed. IDO1 was reported to be upregulated in the tumour cells but not significantly in immune cells. Expression of IDO1 was not dose dependent and there was no significant increase in the CD8^+^ or CD4^+^ effector T cells or tumour-infiltrating leucocytes. Although the combination treatment safety profile indicated that it is tolerable, it did not provide significant improvement to patient outcome as compared to single atezoluzumab therapy.

## Conclusion

Technologic progresses in early diagnostic have significantly reduced BrCa mortality rate through early cancer detection. Moreover, research has revealed the multiple underlying mechanisms of BrCa pathology, leading to the development of various targeted treatments aiming to improve BrCa patient outcomes further. However, treatment for advanced BrCa and/or metastasis remains a challenge. Immunotherapy has been one of the treatments to limit the spread of cancer, and the KP has emerged as a key mechanism for targeted treatments.

In this review, we have presented strong evidence that the KP is dysregulated in BrCa and that several of its enzymes may be potential targets to enhance the anti-tumoral immune response. In particular, the positive outcomes from animal studies have led to multiple clinical trials examining the potential of combining IDO1 inhibitor in combination treatments for cancer patients, including six clinical trials with BrCa patients. However, these combined therapies did not improve patient outcomes as compared to monotherapy. These discouraging results echoed the failed phase I/II clinical trial evaluating epacadostat plus pembrolizumab in malignant melanoma. There were no significant differences between the treatment groups for progression free survival in epacadostat and pembrolizumab arm (*n* = 354) or placebo plus pembrolizumab (*n* = 352) (4.7 months and 4.9 months respectively). In addition to this, treatment-related adverse events were reported in 10% of patients receiving the combination treatment and 9% of patients receiving the placebo [160]. It is noteworthy to mention that this may be due to the absence of an IDO1 status assessment of the tumour prior to patient enrolment as highlighted in a review by Van den Eynde et al. [161]. In the six trials with BrCa patients, only half of the six trials assessed IDO1 status of the tumour during the trial. Therefore, most of the patients who did not respond may not have tumour with IDO1 expression, thus skewing the clinical trial data [162].

There are also some concerns about IDO1 inhibition that would need to be addressed in future studies. The first research question to address is whether a compensatory mechanism is activated when IDO1 is inhibited. Considering that TDO and IDO2 are in the same node of the pathway, inhibition of IDO1 may lead to a feedback mechanism to overexpress TDO and/or IDO2. TDO may likely be the prime substitute enzyme as it is not only expressed constitutively [76, 163] but has also been suggested to replace IDO1 as the driver for the KP during metastases in a tumour metastasis mouse model study [164]. The other concern that requires attention is that IDO1 inhibitor may activate AhR due to its chemical structure [165]. Overactivation of AhR is common in cancer and is associated with poor prognosis [166]. Although the AhR activation by IDO1 inhibitor is weak compared to its natural ligands, this might potentially be a contributing factor to the negative outcome of IDO1 inhibitor clinical trials.

Overall, the KP has been shown to have a strong association with BrCa and may play a role in the pathology of this cancer. An in-depth understanding of the pathway could either lead to better development of KP inhibitors or a monitoring tool for treatment response for BrCa. As demonstrated in a recent publication of a clinical trial examining the combination of Indoximod with checkpoint inhibitors, the combination therapy was well tolerated and showed anti-tumour efficacy in patients with melanoma [167]. Ongoing research may likely show similar promise for BrCa patients.
